# Rickettsial infections: A blind spot in our view of neglected tropical diseases

**DOI:** 10.1371/journal.pntd.0009353

**Published:** 2021-05-13

**Authors:** Jeanne Salje, Thomas Weitzel, Paul N. Newton, George M. Varghese, Nicholas Day

**Affiliations:** 1 Centre for Tropical Medicine and Global Health, Nuffield Department of Medicine, University of Oxford, Oxford, United Kingdom; 2 Mahidol Oxford Tropical Medicine Research Unit, Faculty of Tropical Medicine, Mahidol University, Bangkok, Thailand; 3 Public Health Research Institute, Rutgers, the State University of New Jersey, Newark, New Jersey, United States of America; 4 Laboratorio Clínico, Clínica Alemana de Santiago, Facultad de Medicina Clínica Alemana, Universidad del Desarrollo, Santiago, Chile; 5 Instituto de Ciencias e Innovación en Medicina (ICIM), Facultad de Medicina Clínica Alemana, Universidad del Desarrollo, Santiago, Chile; 6 Lao-Oxford-Mahosot Hospital-Wellcome Trust Research Unit (LOMWRU), Microbiology Laboratory, Mahosot Hospital, Vientiane, Lao PDR; 7 Department of Infectious Diseases, Christian Medical College, Vellore, India; Chengde Medical University, CHINA

## Abstract

Rickettsial diseases are a group of vector-borne bacterial infections that cause acute febrile illness with potentially severe or fatal complications. These vector-borne diseases are prevalent in tropical and subtropical regions worldwide and disproportionately affect poorer communities but are scientifically underrecognized. Despite this, they are not included in the World Health Organization’s list of neglected tropical diseases nor were they mentioned in Peter Hotez’s recent reflections on “What constitutes a neglected tropical disease?” in *PLOS Neglected Tropical Diseases* [1]. Here we present the case that rickettsial infections, as an overlooked cause of morbidity, mortality, and economic losses in marginalized populations, should be recognized as neglected tropical diseases. We describe how this oversight is the result of a number of factors and how it negatively impacts patient outcomes. We then propose measures to address the neglect of rickettsial infections in both scientific research and public health interventions.

## Introduction to rickettsial diseases

Rickettsial diseases (i.e., species within the order Rickettsiales) are caused by a group of vector-borne, Gram-negative obligate intracellular bacteria transmitted by ticks, fleas, lice, and mites. With the availability of molecular diagnostic tools, many new rickettsial species have been discovered [2], and rickettsioses account for the most common emerging/reemerging zoonoses globally [3]. Scrub typhus, probably the world’s most important rickettsial infection in terms of disease burden, occurs predominantly in the geographic area called the “tsutsugamushi triangle” which extends from northern Japan and Eastern Russia in the north to northern Australia in the south and Pakistan in the west causing nearly a million cases per year with an estimated 1 billion people at risk [4,5]. However, there is increasing evidence of the disease being present in other areas including Africa, Middle East and South America [6]. Typical symptoms of rickettsial infections include fever, headache, and sometimes a skin rash. In scrub typhus and some spotted fever group rickettsial infections, an eschar develops at the site of the arthropod bite. Acute complications include pneumonitis, acute respiratory distress syndrome, meningoencephalitis, jaundice, acute kidney injury, myocarditis, and septic shock. The diseases vary in severity, but many can be life threatening if not treated with appropriate antibiotic therapy early in the infection. Rickettsias are vector borne (with the exception of *Neorickettsia sennetsu* which is probably transmitted by consuming infected fish) and possess complex life cycles, which are often only poorly understood. Reservoirs are wild or domestic vertebrates, although some species are mostly or exclusively maintained by vertical transmission within their vector populations.

## The public health burden of rickettsial infections

Neglect of rickettsial infections takes the form of underrecognition (and therefore undertreatment) through a lack of available diagnostics, rather than the absence of an effective specific treatment. This underrecognition hinders an accurate assessment of global disease burden. However, multiple published fever and seroprevalence studies, and associated meta-analyses, attest that rickettsial infections, particularly scrub typhus and murine typhus, constitute a major disease burden in many parts of the world [7,8] as great or greater than many of the diseases already on the World Health Organization (WHO) and *PLOS Neglected Tropical Diseases* lists. Seroprevalence studies in Bangladesh, India, Indonesia, Laos, Malaysia, Papua New Guinea, and Sri Lanka yielded rates for *Orientia tsutsugamushi* exposure of between 9.3% and 51.6% [7,9]. In a review of acute febrile illness studies in India, scrub typhus was found to be the cause of illness in 16.1% to 47.5% of incident patients, and a rate of 96.9% in an investigation of an outbreak of fever of unknown origin in Arunachal Pradesh [7]. The same authors reviewed 39 studies including 91,692 cases of scrub typhus and found reported case fatality rates to range from 0% to 33.3% (median 1.4%). Taken together, these findings suggest a considerable public health burden associated with scrub typhus.

Apart from *Rickettsia rickettsii* (Rocky Mountain spotted fever), which is relatively well studied, and *Rickettsia prowazekii* (epidemic typhus), which is thankfully currently very rare, other *Rickettsia* species are generally less lethal than scrub typhus but are global in their distribution. Because of a paucity of diagnostics, they are also understudied and neglected, but there is evidence that they make a considerable clinical impact. In travelers returning from Africa presenting with febrile diseases, rickettsiosis is the second most frequent cause after malaria [10], though very underdiagnosed in Africa itself [11]. Regional studies from different African countries highlight high exposure rates. In Southwestern Tanzania, for example, 44% to 92% of the population had IgG antibodies against SFG rickettsias [12]. Another study from Senegal found rickettsial DNA in 7% (9/134) of blood samples of patients with non-malarial febrile disease [13]. These data, although rudimentary, suggest that rickettsiosis is a frequent but completely neglected cause of febrile bacterial disease in tropical Africa. Its correct identification and treatment would avoid unnecessary morbidity and the overuse of beta-lactam antibiotics, which are ineffective for rickettsias but contribute to the emerging problem of antimicrobial resistance.

Few neglected diseases, by definition, have accurate burden estimates or accurate databases and maps of incidence and prevalence. Two systems that collate and curate such data are available. Vectormap reports published data on the distribution of humans with scrub typhus and on chiggers but is currently undergoing an upgrade so is offline (http://vectormap.si.edu/Project_ScrubTyphus.htm; http://vectormap.si.edu) [14]. Rickettsia.net has begun collating details for diverse *Rickettsia* and *Orientia* spp. human infections (https://www.rickettsia.net).

### Rickettsial diseases and their association with poverty

With the availability of molecular diagnostic tools, “emerging” rickettsial diseases have been newly recognized in many regions, with areas of endemicity being determined by the presence of the rickettsial-infected vectors [15]. The populations most exposed to these vectors and at highest risk of infection are those living in poverty and with the least access to modern healthcare [16]. There are a number of reasons for this. Many vectors feed on rodents and therefore proximity of living quarters to rodent populations increases the risk of exposure. Stray dog populations are another potential reservoir for rickettsias. These factors are evident in the outbreaks of murine typhus in homeless populations of cities such as Los Angeles [17] or clusters of Rocky Mountain spotted fever in rural populations in Mexico [18]. Agricultural workers are at particular risk of rickettsial infection because of time spent outdoors and proximity to potentially infested animal and vector populations. Children are another risk group due to their increased exposure to certain vectors, e.g., the brown dog tick or rat fleas [18,19]. *R*. *prowazekii*, the causative agent of epidemic typhus, one of the most severe rickettsial infections, can spread between infected people via the human body louse. Outbreaks are typically associated with crowded and unsanitary conditions such as prisons and refugee camps. High-risk groups also include marginalized people in wealthy countries such as drug users or the homeless [20]. With the dire impact of the COVID-19 pandemic on the health, economy, food, and disease security of the global vulnerable population and hazards of civil strife, we risk a return of devastating consequent problems, such as epidemic typhus. Although our epidemiological knowledge of rickettsioses outside the industrialized world is fragmentary, the list of rickettsial pathogens threatening people in poverty in nonindustrialized countries is considerable (Table 1); however, the known spectrum most probably only represents a fraction of the real problem. In rural vulnerable communities dependent on farming, neglected rickettsial diseases are very likely to have a profound chronic negative impact on ability to farm, reducing productivity and hence adding to the burden of adverse events that engender worsening poverty.

**Table 1:** An overview of rickettsial diseases, their vectors, and their known regions of endemicity.

Rickettsial disease	Causative agent(s)	Vector	Geographic region	Risk of exposure
Human granulocytotropic anaplasmosis	*Anaplasma phagocytophilum*	Ticks	North America, Europe, Asia	Rural and agricultural activities
Human monocytotropic ehrlichiosis	*Ehrlichia chaffeensis*	Ticks	Worldwide	Rural and agricultural activities
Sennetsu (glandular fever)	*Neorickettsia sennetsu*	-	Asia	Consuming raw fish?
Spotted fever group rickettsioses	*Rickettsia rickettsii*, *Rickettsia conorii*, *Rickettsia africae*, *Rickettsia parkeri*, *Rickettsia honei*, *Rickettsia australis*, *Rickettsia helvetica*, *Rickettsia japonica* and many others	Ticks	Worldwide	Rural and agricultural activities
Murine typhus	*Rickettsia typhi*	Fleas	Worldwide	Poor living conditions and urban areas
Epidemic typhus	*Rickettsia prowazekii*	Lice	Worldwide	Civil war, refugee camps, prisons, lack of hygiene in cold or mountainous areas
Scrub typhus	*Orientia tsutsugamushi*, *Candidatus* Orientia chuto, *Candidatus* Orientia chiloensis	Mites	Asia, Northern Australia, Chile, United Arab Emirates	Rural and agricultural activities

### Why are rickettsial diseases neglected?

Major reasons why rickettsial diseases have been overlooked by researchers and physicians are that clinical presentation resembles that of other tropical diseases, so clinical diagnosis is difficult or impossible. Laboratory diagnostics are poorly performing and generally unavailable in resource poor settings. This leads to a situation where the known prevalence is underreported, with downstream impacts on funding and public health decisions [21]. In a circular manner, the difficulties in diagnoses have led to a lack of investment which could have enabled the development of improved diagnostic tools and a greater recognition of the incidence of these diseases. In this sense, their recognition as rare and regional diseases is creating a self-fulfilling prophecy. As an example, a comparison of the epidemiology of the mite-borne rickettsial pathogen *Orientia* spp. in 2000 and 2020 shows an expansion from one species endemic in Asia and Australasia (the tsutsugamushi triangle) to at least 3 species with probably worldwide distribution, including South America, Africa, and Middle East [6]. Put simply, these regions were considered scrub typhus free until researchers questioned traditional paradigms and started to investigate [22]. It is likely that the same would be found with other known and as yet unknown rickettsial pathogens. With increasing climate changes contributing to global warming, it is becoming apparent that many of those rickettsial diseases currently restricted to tropical countries will expand their geographical range.

The abovementioned association with poverty is another reason why rickettsial diseases have been neglected, since marginalized communities have a limited voice in setting public health priorities. This is demonstrated by comparison with another vector-borne bacterial infection, Lyme disease, that mainly affects the Global North and has received substantial dedicated funding and a high public profile. Although *Borrelia burgdorferi* s.l., the causative agent of Lyme disease, was only discovered in 1984 and its complex life cycle is still considered as “poorly understood,” the body of knowledge is far larger than for any of the tick, louse, or mite-borne rickettsial infections which have been known about for much longer and are potentially more severe (Table 2).

**Table 2:** Body of knowledge regarding the transmission of different vector-borne pathogens. Numbers represent total PubMed hits for combined All Fields searches.

AND	Known since	“ecology”	“reservoir”	“life cycle”
**“*Plasmodium falciparum*”**	<1950	321	278	1,303
**“*Borrelia burgdorferi*”**	1984	338	402	150
**“*Rickettsia rickettsii*”**	<1950	31	9	9
**“*Rickettsia conorii*”**	<1950	9	21	8
**“*Rickettsia typhi*”**	<1950	16	13	5
**“*Rickettsia africae*”**	1996	10	3	1
**“*Rickettsia prowazekii*”**	<1950	13	10	4
**“*Rickettsia felis*”**	1996	17	21	3
“*Orientia tsutsugamushi*”*	<1950	30	20	10
***”Anaplasma phagocytophilum”***	<1950	113	173	41
***“Ehrlichia chaffeensis”***	1991	13	48	13

*“*Orientia tsutsugamushi*” OR “*Rickettsia tsutsugamushi*.”

Search date: 22 October 2020.

Another challenge specific to rickettsial pathogens is that, as vector-borne diseases, they are studied from multiple angles by biologists, ecologists, veterinarians, and physicians, but the findings are rarely brought together in a connected multidisciplinary process. This means that newly discovered rickettsial organisms such as *Rickettsia parkeri* may be studied by biologists and ecologists for a considerable length of time before they are recognized as human pathogens.

Finally, clinicians in training in many parts of the world are taught that rickettsial diseases are rare and exotic, with teaching on vector-borne diseases primarily focused on parasites such as *Plasmodium*, *Leishmania*, and *Trypanosoma* spp. and on arboviruses such as dengue, zika, chikungunya, and yellow fever. This leads to a bias against consideration of rickettsial pathogens as potential etiological agents of febrile illness in the clinic.

### What are the consequences of their neglect?

There are few infections that are as dangerous but as easy to treat as rickettsioses; therefore, the impact of their neglect on patient health globally is substantial. Late or no treatment leads to morbidity and mortality, while a lack of diagnostic capability leads to a lack of widespread recognition of the problem. This results in missed opportunities for effective and inexpensive interventions as well as targeted preventive measures to control vectors and zoonotic reservoirs.

Another consequence of the misdiagnosis of rickettsial infections is that it leads to inappropriate antibiotic drug use, which contributes to the globally increasing antimicrobial resistance burden.

The underrecognition of rickettsial diseases as important neglected tropical diseases has translated to years of underfunding in these pathogens and their vectors [23]. This has discouraged new researchers from pursuing careers in this area and therefore limited efforts for translational research into diagnostics, vaccines, and treatments.

### Proposed solutions

An increased awareness of rickettsial diseases as neglected tropical diseases among scientists, clinicians, and patients will improve clinical outcomes. An important first step to raising awareness of rickettsial diseases would be their recognition as neglected tropical diseases by the WHO. Initially, this group of diseases included chronic and debilitating illnesses affecting populations living in extreme poverty. However, recently, the criteria were widened and now also include acute infections such as dengue and chikungunya, and other conditions causing acute sickness such as snake bites and rabies. Furthermore, the recent strategy suggests a less disease-specific and more integrated intervention-centered approach, including vector control and One Health strategies [24]. Considering these new criteria, in our opinion, rickettsial diseases are a straightforward candidate for inclusion and have so far been simply overlooked by the WHO. As explained above, rickettsioses fulfill 3 of the 4 suggested criteria: (1) they cause important morbidity and mortality within populations living in poverty; (2) they primarily affect populations living in tropical and subtropical areas; and (3) they are relatively neglected by research [24]. The list of neglected tropical diseases recognized by *PLOS Neglected Tropical Diseases* is based on broader criteria than the WHO list and more than doubles the number of named diseases considered to be neglected tropical diseases. To be on the *PLOS Neglected Tropical Diseases* list, diseases need to “represent prevalent, high disease burden, human illnesses” [1]. Rickettsial infections vary in incidence and disease burden, ranging from high incidence but relatively low morbidity (African tick bite fever), through low incidence but high morbidity and mortality (epidemic typhus), to infections such as scrub typhus which are widespread, common, and have a significant mortality [7,8]. An explicit criterion for adding a disease to the list is “the availability of disease burden estimates for that specific condition, and if that burden occurs in resource-poor settings.” We consider rickettsial infections to be neglected tropical diseases in terms of incidence and burden and propose that both WHO and *PLOS Neglected Tropical Diseases* lists of neglected tropical diseases be expanded to include rickettsial infections.

The complex ecology of rickettsial infections requires a One Health approach. In this, healthcare is defined by an interconnected system of humans, animals, and plants within their environment, and scientific research and public health interactions are viewed through this lens. Greater communication and synergy between veterinarians, entomologists, ecologists, clinicians, geospatial, temporal, and niche analysists and microbiologists would enable such an approach in the study of rickettsioses. Specifically, funds for interdisciplinary research on rickettsial infections, the establishment of laboratory partnerships, and the organization of shared meetings between disparate communities would support these efforts. Rickettsioses transmitted by “neglected vectors” such as mites and fleas, which have been scientifically almost abandoned for decades [23], could benefit rapidly from such increased scientific attention.

Finally, the development of sensitive, specific, affordable, and easy-to-use diagnostics for pan-rickettsial infections would have an outsized impact on the field. This would enable a much more detailed understanding of the global distribution of these diseases, which would in turn spur and direct government funds and inform public health priorities. It would enable physicians to routinely screen for rickettsioses in patients with “undifferentiated” febrile illnesses, and ultimately lead to improved outcomes for patients. Designing a comprehensive surveillance system in endemic areas for rickettsial diseases coupled with distribution of affordable rapid diagnostics which can detect the disease early will lead to a significant reduction in morbidity and mortality.

Rickettsial infections are unusual among neglected tropical diseases in causing life-threatening illness that is easy to treat if appropriately recognized in a timely manner. For this reason, simply raising awareness of their prevalence has the potential to substantially reduce patient mortality and morbidity in endemic areas at relatively little cost. It is for this reason that we strongly support the inclusion of rickettsial diseases in both the official WHO and *PLOS Neglected Tropical Diseases* lists of neglected tropical diseases, which will lead to increased recognition by policymakers, funders, healthcare workers, and scientists.
